# Bridging may help young female Tibetan macaques *Macaca thibetana* learn to be a mother

**DOI:** 10.1038/s41598-018-34406-7

**Published:** 2018-11-02

**Authors:** Dao Zhang, Dong-Po Xia, Xi Wang, Qi-Xin Zhang, Bing-Hua Sun, Jin-Hua Li

**Affiliations:** 1 School of Resources and Environmental Engineering, Anhui University, Hefei, 230601 Anhui China; 2 School of Life Sciences, Anhui University, Hefei, 230601 Anhui China; 3 School of Life Sciences, Hefei Normal University, Hefei, 230601 Anhui China

## Abstract

Attraction to infants is a common feature of non-human primates. Frequent affiliative male-infant interactions have been observed in many multimale, multifemale groups of macaques, including a behaviour termed ‘bridging’ in which two male macaques simultaneously lift an infant. This behaviour has been suggested to serve as a positive affiliative interaction between the adult or subadult males. Female macaques display bridging in the same manner as males, but the function of this behaviour to females remains unknown. In this study, we examined evidence for the function and evolution of bridging in female Tibetan macaques within the framework of three hypotheses: the learning to mother, a side-effect of selection for appropriate maternal care, and alliance formation hypotheses. Our results showed that subadult females initiated more bridging than adult females. Females preferred to use infants for bridging when the infants were less than four weeks old. Female frequency of received bridging with higher-ranking females was not significantly different from their frequency of received bridging with lower-ranking females. Bridging frequency was not significantly different between dyads composed of related and unrelated females. Additionally, post-bridging grooming frequency was significantly higher than nonbridging grooming interactions, suggesting a social function for bridging. The results of our study supported the ‘learning to mother’ hypothesis, suggesting that bridging among female intrasexual dyads is a multi-functional, complex and differential evolutionary process.

## Introduction

Alloparental care is common among mammals^1^. Typically, recipients of this care and/or their parents experience enhanced survival, and caregivers benefit via increased production of non-descendant kin^2^. Previous studies have demonstrated that alloparental interactions come in different forms and the maternal response to infant handling is variable in non-human primates^3^. For example, in capped langurs, *Presbytis pileata*, newborn infants under one month old spend nearly the same amount of time with alloparental females as with their own mothers^4^. Moreover, the phenomenon of young females with no infant in vervet monkeys, *Chlorocebus aethiopssabaeus*, holding or carrying newborn infants is explained by alloparental investment^5^. However, in macaques and baboons, attraction to infants is minimized to direct care, which is known as ‘infant handling’^3^. Most macaque and baboon mothers receive virtually no direct help in feeding, carrying, grooming, or protecting their offspring from predators^6^. It seems unlikely that infant handling by alloparent has direct benefit to the infants’ development and/or reduces the parental investment of the mother.

Like other handling behaviour involving infants, bridging is a behavioural phenotype seen in several macaque species (i.e. Barbary, *Macaca sylvanus*^7^; Tibetan, *M. thibetana*^8^; stump-tailed, *M. acrtoides*^9^; long-tailed, *M. fascicularis*^10^; Assamese, *M. assamensis*^11,12^; and bonnet, *M. radiata*^13^). ‘Bridging’ is defined as two handlers that simultaneously hold or pick up the same infant. The handlers often touch the infant’s genitalia, showing typical facial expressions accompanied by teeth-chattering^8,14^.

Previous studies have shown that bridging has different functions among handlers and infants depending on sex, social rank, and infant age^15^. There is also variation in a bridging partner and the type of infant^16^. Male preferences for infants to bridge with may reflect their social relationship with the mother and may predict future mating opportunities^17–19^. Males initiate more bridging interactions with relatively higher-ranking males than with lower-ranking males to buffer aggressive pressure from higher-ranking males^8,13,20,21^. Bridging can be also considered as a triadic association with direct and indirect impacts on the social relationship between the two adults who handle the infant^12,20^. Additionally, bridging can be a predictor of the social bonds between two males, between a male and a female^17,19^, and between females as well

Several hypotheses have been proposed to explain the potential adaptive function of infant handling. The ‘learning to mother’ hypothesis states that infant handling enhances maternal skills in subadult females who handle the infant^22^, which increases their subsequent reproductive success. In female primates, the infant handling may be explained as a side-effect of selection for appropriate maternal care if females who are highly responsive to infants make good mothers^23,24^. This hypothesis predicts that maternal solicitude is critical for a female to successfully rear an infant; therefore, they will be interested in younger infants, even other females’ infants after they had given birth to their own infants. Females spend much of their time in proximity to maternal kin, so females may handle related infants at higher rates than they handle unrelated infants^23^.

Alternatively, the ‘alliance formation’ hypothesis^3,19,25–27^ suggests that infant handling benefit the handler by establishing a profitable relationship with the infant’s mother. According to this hypothesis, females should initiate more bridging with higher-ranking females than with lower-ranking females, and handlers should improve their relationships with an infant’s mother as a consequence of infant handling.

Bridging, as a behavioural interaction, performs clear and critical social functions in non-human primate societies. However, most studies to date have focused on bridging between male intrasexual and intersexual dyads, while the function and evolution of bridging between female intrasexual dyads remains unclear.

**Figure 1:**
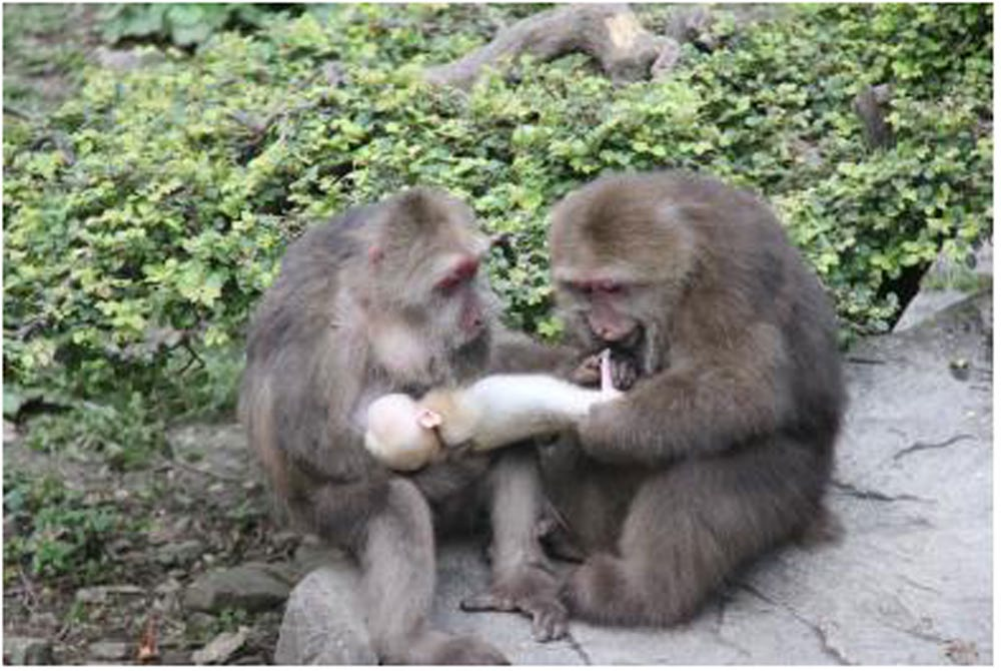
Bridging behaviour in Tibetan macaques between two females. The left female is the infant’s mother.

This study aimed to examine the function of bridging among female Tibetan macaques. Like other macaque species, Tibetan macaques live in multimale, multifemale social groups. The groups are female philopatric with male dispersal; thus, females remain in their natal groups throughout their lives^14^. *M. thibetana* is a seasonal breeder, with the birth season lasting from February to June^14^. Similar to other non-human primates^3^, group members show an intense interest in infant handling. Both males and females participate extensively in infant handling through bridging^14^ (see Fig. 1). Bridging among adult males can be considered as a triadic awareness of partner choice not only to reduce the probability of an aggressive response from dominant males, but also to facilitate male intrasexual or intersexual social bonds^8,19^. However, it is common to find adult females using infants to conduct social bridging with other adult females, one of which being the infant’s mother. It is still unknown why bridging exists among adult females.

The aim of this study was to test the following hypotheses, using the following predictions:

Hypothesis 1: Bridging enhances young female maternal skills.

1a. Nulliparous females participate in bridging more frequently than parous females.

1b. Nulliparous females who participate the most in bridging are more likely to have their firstborn infant surviving.

Hypothesis 2: Bridging is a side-effect of selection for appropriate maternal care.

2a. Females most often use the youngest infants for bridging.

2b. Adult females initiate bridging as often as subadult females.

2c. Females receive bridging from related individuals more often than from unrelated individuals.

Hypothesis 3: Bridging is a form of alliance to strengthen dyadic social relationships.

3a. Females initiate more bridging with higher-ranking females than with lower-ranking females relative to their own rank.

3b. After giving birth, females initiate more bridging.

3c. Bridging strengthens dyadic social affiliation, such as grooming, which occurs more often when bridging has occurred than when bridging did not occur between two females of proximity.

## Results

### Patterns of bridging

Hourly rates of infants used for bridging were based on each focal mother. This value ranged from 0.6 to 28 as five of the infants died during the study period. A total of 248 acts of female-female bridging were observed. Most of the bridging was of Type 1: a female approached another female who was with her infant, and used the infant for bridging (77.4%, n = 192). This was followed by Type 2: the infant’s mother approached another female and initiated bridging using her own infant (22.4%, n = 51). Type 3: other cases, e.g. a female holds another female’s infant and approaches another female; neither are the infant’s mother, only constituted 0.2% (n = 5) of total bridging. Bridging was unevenly distributed across females (Kolmogorov-Smirnov test: *t* = 3.587, *P* = 0.004, *n* = 13, *df* = 12). Two of the 13 females were not observed to engage in bridging. Bridging frequency amounted to 0.69 acts/h on average (SE = 0.05, range 0–2.25, n = 13).

### Age differences in bridging

**Figure 2:**
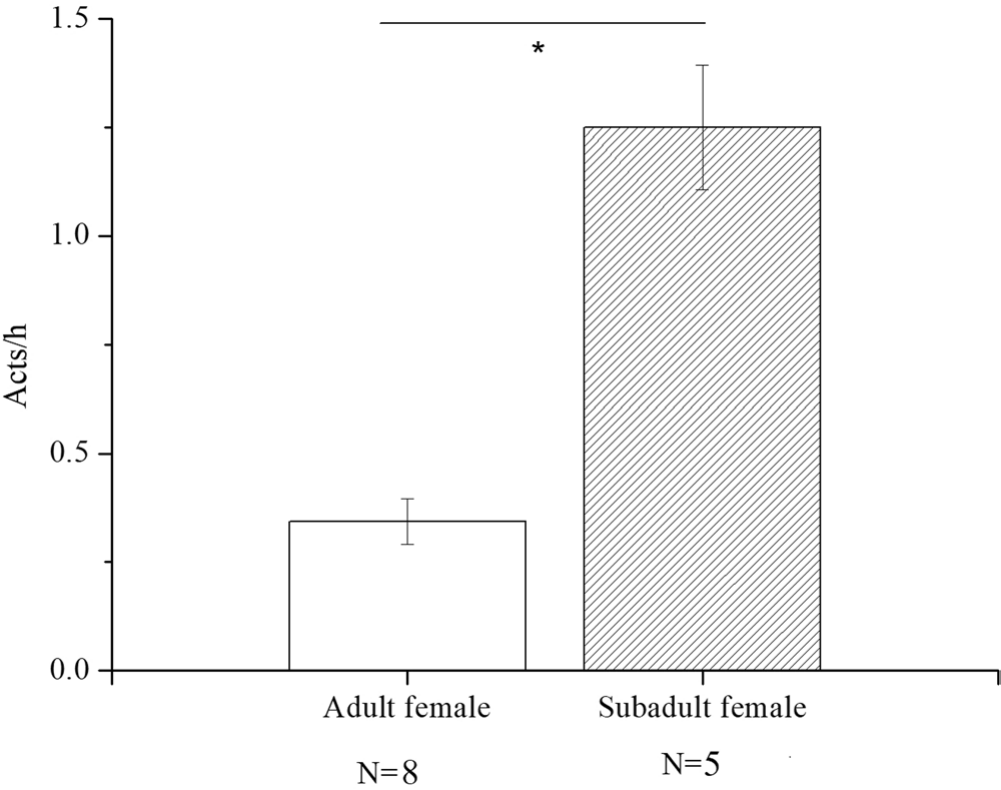
The mean (±SE) frequency (acts/h) of bridging in females initiated by adults and subadults.

On average, mothers received bridging by subadult females (mean ± SE: 1.25 ± 0.14 acts/h) more frequently than by adult females (mean ± SE: 0.34 ± 0.05 acts/h) (Wilcoxon signed-ranks test: *Z* = −2.202, n_1_ = 8, n_2_ = 5, *P* = 0.028, see Fig. 2).

### Effect of bridging on firstborn infant survival

Only 2 of the 5 (40%) firstborn infants survived after 3 months of age, meanwhile 7 of 9 (78%) infants of the multiparous mothers survived first three months of life. In total only 4 infants were still alive at the end of the study period, 2 firstborn infants and 2 infants of multiparous mothers. On average, the two subadult females that successfully reared their firstborn infants in the first 3 months bridged more often than the three females whose infants died (mean ± SE: survived: 1.98 ± 0.38 acts/h; died: 0.38 ± 0.18 acts/h).

### Effect of infant age

**Figure 3:**
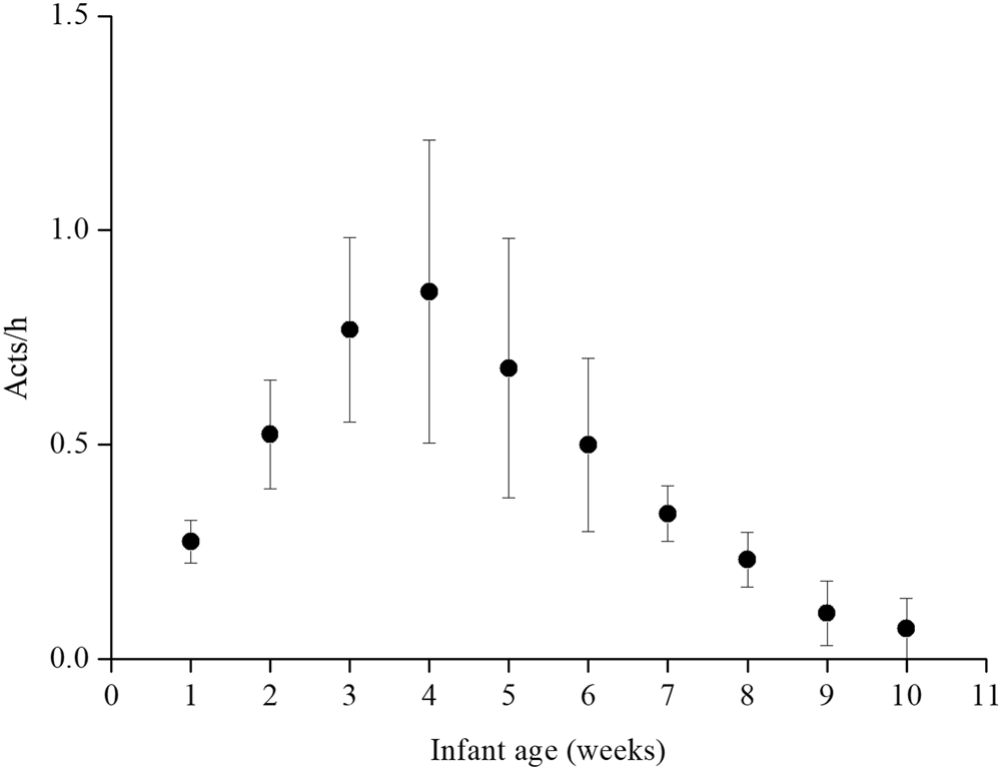
The effect of infant age on the mean (±SE) frequency (acts/h) of bridging in females.

Infants were more likely to be used in bridging when they were 4 weeks old. The hourly rate of bridging declined as infants matured (see Fig. 3). In their first four weeks of life, infants were used in bridging on average 0.86 acts/h by adult and subadult females. At nine weeks of age, the average rate of bridging dropped to 0.11 acts/h.

### Effects of kinship

**Figure 4:**
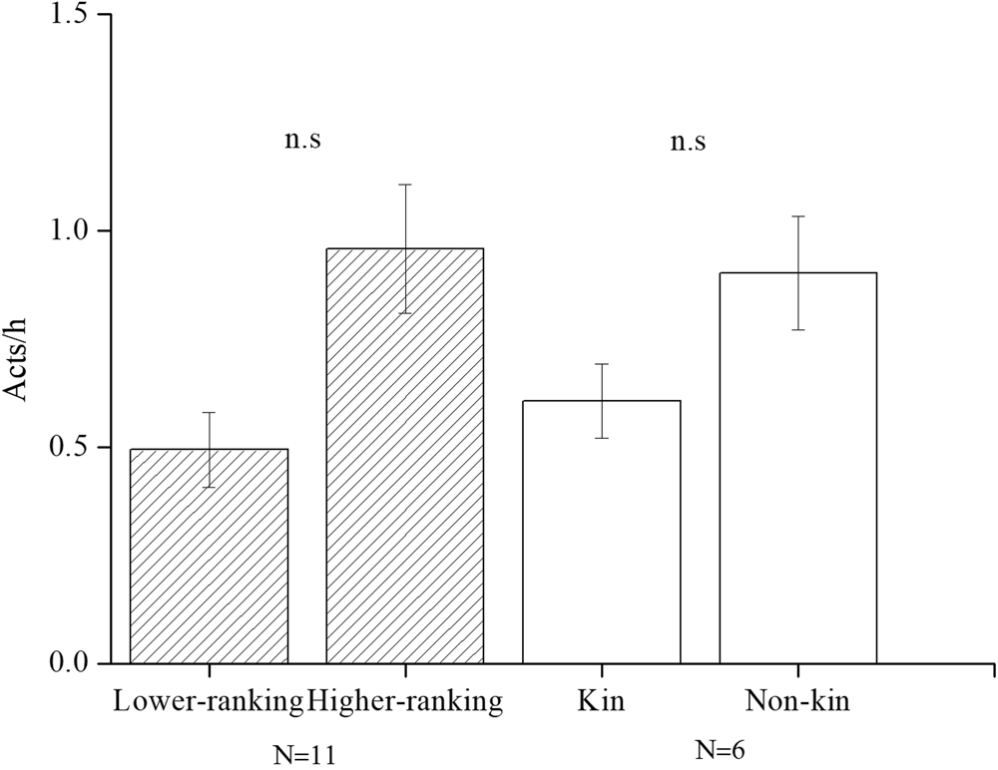
The mean (±SE) frequency (acts/h) of bridging in females initiated by lower and higher-ranking females and kin and non-kin females.

The rate of bridging between maternal kin (mean ± SE: 0.61 ± 0.09 acts/h) was not significantly different from the rate of bridging with non-kin (mean ± SE: 0.90 ± 0.13 acts/h) (Wilcoxon signed-ranks test: *Z* = −1.183, *P* = 0.237, n = 6, see Fig. 4).

### Effects of dominance rank

**Figure 5:**
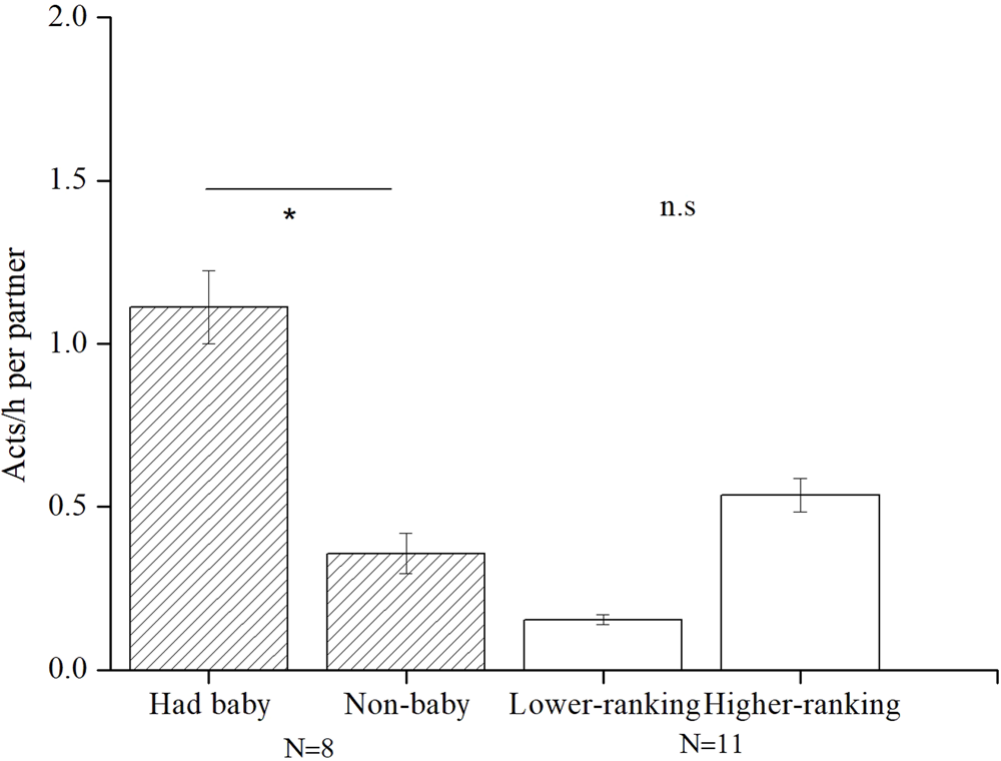
Mean (±SE) (acts/h) bridging initiated by the birth condition and the rank.

The rank of the mother did not influence the frequency of bridging received (Wilcoxon signed-ranks test: *Z* = −1.363, *P* = 0.173, n = 6). On average, mothers received 0.49 ± 0.09 acts/h of bridging from lower-ranking females, and 0.96 ± 0.15 acts/h from higher-ranking females (Fig. 4). Higher ranking females had higher rates (0.54 ± 0.13 acts/h) of bridging and initiated more bridging with subordinate lower ranking females than with higher ranking ones (0.15 ± 0.04 acts/h); however, this difference was not significant (*Z* = −1.599, n = 11, *P* = 0.110, see Fig. 5).

### Differences in initiating bridging after birth, affiliative behaviour after bridging, and rank variation

**Figure 6:**
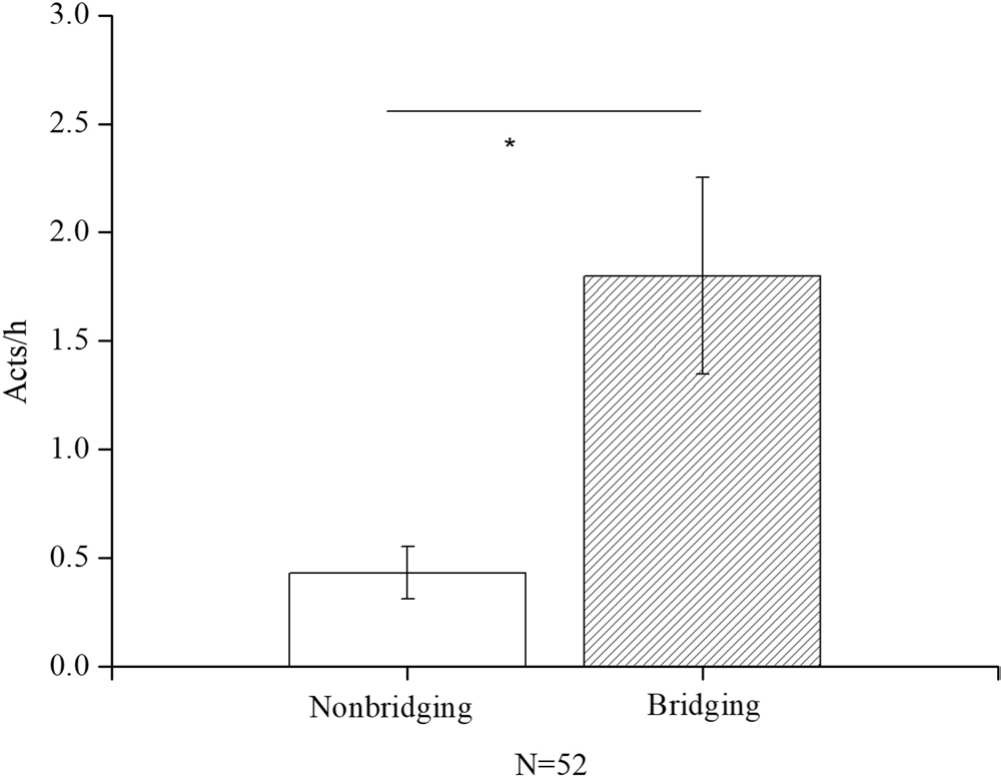
Mean (±SE) (acts/h) of grooming frequency between females of close proximity after bridging and nonbridging behaviour.

Females were more likely to initiate bridging after they gave birth. The rate of bridging initiation before and after a female gave birth differed significantly (*Z* = −2.003, n = 8, *P* = 0.045). The frequency of bridging increased from 0.036 acts/h before birth to 1.10 acts/h after birth (see Fig. 5). Additionally, grooming occurred more frequently after bridging than when no bridging occurred between two females in close proximity (Spearman’s rank correlation, *rs* = 0.45, n = 52, *P* = 0.017, Fig. 6). Social ranks among females showed an upward/downward trend from 2014 to 2015, particularly for subadult females after their first birth. YCY and TXH increased their rank from 5 to 3 and 6 to 5, respectively, and displayed more bridging (2.25 acts/h and 1.71 acts/h, respectively) than THY (0.25 act/h), whose rank decreased from 10 to 13 (Table 1).

**Table 1:** Group composition of female and infants including birth and death dates during the study period.

ID	Age	2014				2015			
		Rank	Infant’s birth date	Infant’s death date	Focal time (hours)	Rank	Infant’s birth date	Infant’s death date	Focal time (hours)
YH	11	1	2014/6/4	2014/10/11	15(14)	1	2015/4/19		14(4.6)
1411YM	24	2			15	4			14
TH	11	3	2014/6/7	2014/10/10	15(14)	7	2015/5/19	2016/3/23	14
YXX	4	4			15	2	2016/2/?*	2016/2/?	14
YCY	5	5			15	3	2015/2/15*		14(10)
TXH	5	6			15	5	2015/4/11*		14(6)
TXX	6	7			15	9			14
HH	11	8			15	6	2015/2/20		14(9)
TR	10	9	2014/3/22	2014/8/11	15(14)	8	2015/3/21	2015/8/15	14(14)
THY	5	10			15	13	2015/4/9*	2015/4/20	14(0.6)
TRY	5	11	2014/2/24*	2014/5/3	15(14)	10	2015/4/2	2015/6/2?	14(8)
TT	23	12			15	11	2015/8/5	2015/11/1	14
YZ	22	13			15	12			14

Note: The female ages were that of age in the year 2014. *Refers that of infants was the firstborn infant. Figures in parentheses represent observation time females had infants within 10 weeks.

## Discussion

In baboons and many macaque species^3,25,28^, subadult females are more likely than adult females to engage in dyadic infant handling. In this study, Tibetan macaque bridging were initiated by nulliparous females more frequently than parous females. Consistent with the dyadic infant handling observed in bonnet macaques^23^, white-headed langurs^29^, and capped langurs^4^, Tibetan macaque females preferred to use younger infants for bridging (when the infants age was less than four weeks). Dominance rank had no significant impact on bridging, which differs from the infant handling pattern seen in male Tibetan macaques^8^ and female Bonnet macaques^23^ but is consistent with the behaviour of female Barbary macaques^25^. Maternal kinship did not affect female Tibetan macaque bridging patterns. In general, the ‘learning to mother’ hypothesis was supported.

The fact that immature females were more likely to initiate bridging supports the ‘learning to mother’ hypothesis. This pattern is consistent with previous studies of infant handling in baboons and some macaque species^3,25,28^, as infants were always handled gently^23,29^. The ‘learning to mother’ hypothesis predicts that contact with infants promotes the caring skills of subadult females. In fact, unlike in dyadic infant handling, in bridging the partners touch and lick the infants at the same time as though mimicking the mother. We cannot know how this affects the infants, as the same touching and sucking behaviour was usually observed in mothers towards their own infants. This mimetic behaviour may effectively promote the mothering skills of subadult females. In addition, the ‘learning to mother’ hypothesis predicts that immature females that have more contact with infants will have increased survival of firstborn infants relative to conspecifics who had less contact with infants. For example, in Barbary macaques^25^ and bonnet macaques^23^, subadult females that engaged in more infant handling, had higher survival of their firstborn infants. In this study, although this is an anecdotal finding, subadult female Tibetan macaques whose firstborn infants survived had higher rates of bridging initiation.

The ‘side-effect of selection for appropriate maternal care’ hypothesis predicts that adult and subadult females handle infants at the same rates and that the rates decline as the infants grow older. In our study, although the use of Tibetan macaque infants for bridging declined when the infants were four week old, adult and subadult females did not prefer to use the youngest infants for bridging, and the subadult females initiated more bridging than adult females. A side-effect of the ‘selection for appropriate maternal care’ hypothesis also predicts that infant handling provides appropriate maternal care, and infants are handled at higher rates by maternal kin than non-kin. In this study, even though participants touched the infants gently, the choice of bridging partner was not related to maternal kinship. Therefore, these data do not support this hypothesis.

In male Tibetan macaques, bridging has always been connected to the facilitation of affiliative interactions by initiating frequent social grooming and promoting the avoidance of potential aggression in the group (agonistic buffering)^8^. Paul & Kuester^25^ also report that mothers in Barbary macaques are often recipients of triadic interactions. If so, lower-ranking females should bridge more with infants of higher-ranking mothers. However, there was no difference in the rate of bridging initiation between interactions initiated by a higher-ranking female and interactions initiated by a lower-ranking female. Additionally, we never observed females bridging in agonistic contexts. Therefore, this hypothesis was not supported by these data.

Similar to male Tibetan macaques, there was more frequent grooming after bridging, possibly serving to increase the participants’ social rank. Similar to Barbary macaques^30^, infants can be viewed as social tools that facilitate networking among individuals. Since the interaction has very little risk to infants, mothers may become tolerant of this behaviour and less possessive of their infants. Consequently, mothers may strengthen bonds with group members, test social bonds, and ensure their infants develop relationships with other females^31,32^.

In Barbary macaques, adult males spend significantly more time with infants than adult female or juvenile infant handlers and have the highest triadic interaction rates^15^. Contrary to male Barbary macaques who switch to newborn infants each birth season, male Tibetan macaques used infants even when they are more than 1-year-old^16^. Similarly, female Tibetan macaques engage in bridging extensively, but prefer to use younger infants and the triadic association usually includes the infant’s mother as a bridging partner. This difference in timing and partners may indicate different functions of bridging between males and females.

Finally, as infant handling in Tibetan macaques, female bridging may have evolved as an adaptation (e.g. ‘learning to mother’) and an infant care function. However, rather than typical dyadic interactions between adults and infants, bridging is a triadic behaviour. Bridging in Tibetan macaques is a ritualized form of behaviour and may also be considered to have a social function. Taken together, our results show that bridging behaviour among female intrasexual dyads is a multi-functional and complex differentiation process; thus, a single hypothesis cannot explain the function and evolution of this behaviour occurred among intrasexual and intersexual dyads within a social group. Future work need to pay greater attention to the behavioural strategies used by adult males, adult females, and subadults on the differentiation of form and function of bridging with the development of infants.

## Methods

### Study site and subjects

We conducted the study at Mt. Huangshan, Anhui Province, China. The reserve is a World Cultural and Natural Heritage site that is well-known as a tourist destination and research site for the study of Tibetan macaques, *M. thibetana*. The research site is described in further detail elsewhere^33^. The study group, Yulinkeng A1 (YA1), has been observed and monitored by researchers since 1986, providing known individual identities and maternal lineages^34^. The study groups are habituated to people (i.e. from <1 m) and receive modest daily provisioning of 3–4 kg of corn per group (approximately 60 g/individual) by reserve staff to maintain their presence at designated tourist-viewing sites.

The study took place from 2013 to 2015. At the beginning of the study, the group consisted of 41 individuals including seven adult males, eight adult females (>4 years), and five subadult females (3–4 years). Four infants were born in 2014, nine were born in 2015, 2 infants were born after observations were done and thus not considered in our analysis. (infant details are shown in Table 1).

### Behavioural observations and analysis

Using focal animal sampling methods^35^, we set 20 min focal sample and collected behavioural data from 10 August 2013 to 28 September 2014 and 7 March to 12 May 2015. We studied all adult females (N = 8) and subadult females (N = 5) group members. For each females 29 h of observation time were obtained (15 h in 2014 and14h in 2015). All subjects were individually recognized based on distinctive physical features, such as facial or body characteristics. Focal animals were randomly selected, and if we failed to find the focal individual, we observed the next individual on the list and returned to the previous individual when he/she reappeared^36^. Data were collected using a digital voice recorder (version: Sony ICD-AX412F) and by hand by one observer (Zhang). Depending upon the time of year, our daily observations began at approximately 0700–0800 and ended at 1700–1800.

All observational work was approved by the Institutional Animal Care and Use Committee of the Anhui Zoological Society (permit number BH20131202). All experiments were performed in accordance with the approved guidelines and regulations.

We defined bridging following Li^14^ and Ogawa^8^ as: ‘two females sat facing each other, one female pulled up the infant’s shoulder, another female pulled up its hip, and the infant lay on its back, forming a bridge between them’. While raising the infant, one or both females often sucked or touched the infant’s penis or genital area with the expression of teeth-chattering. When bridging occurred, mothers never let their infants be completely taken away from them.

We classified an individual as an initiator when a female approached another female before body contact, and a receiver as a female who was approached by another female before contact. The sequence of bridging was classified into three types: Type 1: a female approached another female who was with her infant and used the infant to bridge. Type 2: the infant’s mother approached another female and initiated bridging using her own infant. Type 3: other cases, e.g. a female holds another female’s infant and approaches another female, neither are the infant’s mother.

In addition to bridging we recorded all occurrences of aggressive and affiliative interactions between the focal animal and any member of the group. Aggressive interactions were defined as an individual threatening, chasing, slapping, grabbing, or biting another individual. Submissive behaviours were scored when an individual showed fearful interactions, such as a fear grin, cower, mock leave, avoid, flee, or scream^37^. Winer/loser matrices of these interactions were scored *ad libitum* and these data were used to calculate David’s Score for determining dominance relationships^38^. The larger the David’s Score is, the higher the social rank of the individual. Grooming was defined as any act in which a macaque (groomer) used its hand or mouth to touch, clean, or manipulate the fur of another individual (recipient) for a continuous period lasting at least 5 seconds^36^.

Maternal rejection (mother mouthing, threatening, and pushing the infant who attempts to attain, maintain, or regain contact) of the infant was first observed when the infant was 11-weeks-old and continued thereafter^39^. Therefore, we only used data for bridging behaviour occurring in the first 10 weeks of an infant’s life. Group members within the same matriline were treated as kin in the analysis. In this study, the same matriline were mother-daughter and sister. Group members outside of a matriline were treated as non-kin. When we compared rates of bridging in rank lineages, the highest and lowest-ranking lineages were excluded^23^.

We used a Mann-Whitney U test to examine differences in bridging rates between females whose firstborn infants survived and females whose firstborn infants died in the first three months of life. A Wilcoxon signed-ranks test was used to determine differences in all other variables. All analyses were two-tailed, with alpha set at 0.05, and were conducted using SPSS 13.0 software.

## Data Availability

All data generated or analyzed during this study are included in this published article.
